# Structured respiratory physiotherapy protocol for resolution of atelectasis in pediatric intensive care

**DOI:** 10.1016/j.clinsp.2024.100494

**Published:** 2024-09-21

**Authors:** Patrícia Aparecida Silva Camassuti, Cíntia Johnston, Werther Brunow de Carvalho, Michele Luglio, Orlei Ribeiro de Araújo, Brenda Morrow

**Affiliations:** a Postgraduate Program in Pediatrics, Department of Pediatrics, Universidade de São Paulo, São Paulo, SP, Brazil; b Department of Pediatrics, Universidade de São Paulo, São Paulo, SP, Brazil; c Children's Institute, Universidade de São Paulo, São Paulo, SP, Braziil; d Institute of Pediatric Oncology, Support Group for Adolescents and Children with Cancer (GRAACC), Universidade Federal de São Paulo, São Paulo, SP, Brazil; e Department of Paediatrics and Child Health, University of Cape Town: Rondebosch, Western Cape, ZA, South Africa

**Keywords:** Pediatrics, Respiratory physiotherapy, Pulmonary atelectasis, Ultrasound, Mechanical ventilation

## Abstract

Children are at higher risk of atelectasis due to their anatomical and physiological particularities. Several physiotherapy techniques are used to treat atelectasis, but only four studies cite methods in pediatric patients undergoing Invasive Mechanical Ventilation (IMV). The objective of this study was to evaluate the Structured Respiratory Physiotherapy Protocol (SRPP) for airway clearance and lung reexpansion for infants on IMV with atelectasis. This is a prospective study including 30 infants (mean ± standard deviation age 8.9 ± 8.0 months; weight 7.5 ± 3.0 kg; BMI 15.8 ± 1.6 kg/cm^2^ and IMV duration 7.7 ± 4.3 days). The sample was randomized into a Control Group (CG), which received routine physiotherapy, and an Intervention Group (IG), submitted to SRPP (postural drainage, mechanical thoracic vibration, manual hyperinflation, stretching of the accessory respiratory muscles, and functional positioning). Both groups were evaluated before and after physiotherapy for respiratory effort using the Wood Downes Score (WD) and pulmonary aeration using lung ultrasonography (Lung Ultrasound Score ‒ LUS). The outcome of the intervention was evaluated by the magnitude of the effect by the Hedges' g test [(small (0.2 < Hedges' g < 0.5), moderate (0.5 < Hedges' g < 0.8) and large (Hedges' g > 0.8) effects]. There were large within-group effects on the reduction of WD in the CG after intervention in both the CG (Hedges' g = -1.53) and IG (Hedges' g = -2.2). There was a moderate effect on LUS reduction in the CG (Hedges' g = -0.64) and a large effect on IG (Hedges' g = -1.88). This study has shown that the SRPP appears to be safe and may be effective in improving airway clearance and lung reexpansion in children on IMV with atelectasis.

## Highlights

  • Children are at higher risk of developing atelectasis.
  • Imaging studies have increased specificity in the diagnosis of atelectasis.
  • The use of an ultrasound score can quantify pulmonary aeration.

## Introduction

Developing lungs are predisposed to collapse.^1^ Atelectasis is one of the most frequent pulmonary complications in children undergoing Invasive Mechanical Ventilation (IMV).^2^

Children are at higher risk of atelectasis, owing to both obstruction and dynamic airway collapse, due to their distinct anatomical and physiological features. These predisposing features include smaller diameter, less well-supported airways; flexible/compliant chest walls with relatively less compliant lungs; and limited, developing collateral ventilation channels.^3^

In Pediatric Intensive Care Units (PICUs), more than 20% of patients require IMV.^4^ The implementation of protective ventilation strategies with the use of low tidal volumes may, in some cases, contribute to the development of atelectasis secondary to insufficient inflation of the alveolar units.^4^

In addition, infection of the lower respiratory system is one of the leading causes of mortality in children under the age of five.^5^ During Lower Respiratory Tract Infections (LRTI), mucociliary clearance may be impaired by increased inflammation and/or overload due to excessive mucus production, with consequent impairment of ciliary function, predisposing these patients to secondary complications such as pulmonary atelectasis.^6^

Lung collapse may cause or exacerbate increased work of breathing, hypoxemia, hypercapnia, and acute respiratory failure (mild, moderate, or severe).^7^ Long-term complications of unresolved atelectasis include the development of bronchiectasis and chronic lung disease. Early recognition and management of atelectasis are therefore essential to hasten resolution, avoid adverse short and long-term sequelae, and optimize clinical outcomes such as PICU and hospital length of stay and mortality.^8^

The signs and symptoms of pulmonary atelectasis are often nonspecific, however, the application of imaging technologies has increased diagnostic sensitivity and specificity.^9^ Although chest X-Ray is still the gold standard for diagnosing atelectasis, due to its cost and effectiveness,^10^ it has significant limitations including patient exposure to ionizing radiation, relatively low sensitivity in detecting inflammatory lung lesions, low negative predictive value, and discrepancies in interpretation among specialists.^11^

In view of the limitations of radiography, the use of Point of Care Lung Ultrasound (POCLUS) has been proposed as an additional tool for identifying and monitoring pulmonary atelectasis in children. Among the advantages are the low associated financial costs; clinical utility of trained healthcare professionals being able to easily and quickly perform the test at the bedside; and avoidance of harmful ionizing radiation, allowing multiple repeated scans if needed to determine progression and/or response to therapy.^12^

The interventions used in the treatment of pulmonary atelectasis in intubated children are limited and, to date, there are no clinical trials that identify the most efficient treatment for the resolution of pulmonary atelectasis in pediatrics and neonatology.^9^

Chest physiotherapy in patients with pulmonary atelectasis is a minimally invasive treatment that aims to maintain or improve airway patency by removing obstructive secretions, reducing airway resistance, promoting gas exchange, and decreasing the work of breathing.^13^

Although respiratory physiotherapy interventions include several techniques for the treatment of atelectasis, there are only four studies^14^, ^15^, ^16^, ^17^ that cite different methods of respiratory physiotherapy aimed at resolving atelectasis in pediatric patients on IMV. However, none of them are randomized controlled trials and none use standardized, protocol-driven interventions.

Therefore, the main objective of this study was to develop and analyze the clinical and imaging effects of a Structured Respiratory Physiotherapy Protocol (SRRP) for airway clearance and lung reexpansion in children on IMV diagnosed with unilateral pulmonary atelectasis.

## Materials and methods

### Study design and population

This was a prospective randomized controlled clinical trial, registered in the Brazilian Clinical Trials Registry (ReBEC): RBR-106bhfwy, carried out in the PICU of the Menino Jesus Municipal Children's Hospital (HMIMJ), in São Paulo, from October 2020 to March 2022. The study was approved by the Research Ethics Committee of the University of São Paulo School of Medicine (FMUSP) and by the Research Ethics Committee of the HMIMJ (opinion 3,689,413). The study started after its approval.

The study included 30 infants (age: 28 days to 24 months) on IMV for a period greater than or equal to 12 hours through an orotracheal cannula, diagnosed with pulmonary atelectasis by a pediatric intensive care physician through clinical examination and imaging (chest X-Ray and POCLUS), whose legal guardians had authorized the child's participation in the study through the Free and Informed Consent Form (ICF). Exclusion criteria included: patients with bilateral atelectasis; any type of air leak syndrome; pulmonary hemorrhage; presence of diseases presenting with bone fragility; rib cage and/or pulmonary contusions; subcutaneous pacemakers; treatment with anticoagulants for more than 72 continuous hours; hemodynamic instability; thrombocytopenia (platelet count < 50,000); presence of an intercostal chest drain; underlying neuromuscular or cardiac diseases and presence of spinal deformities.

**Fig. 1:**
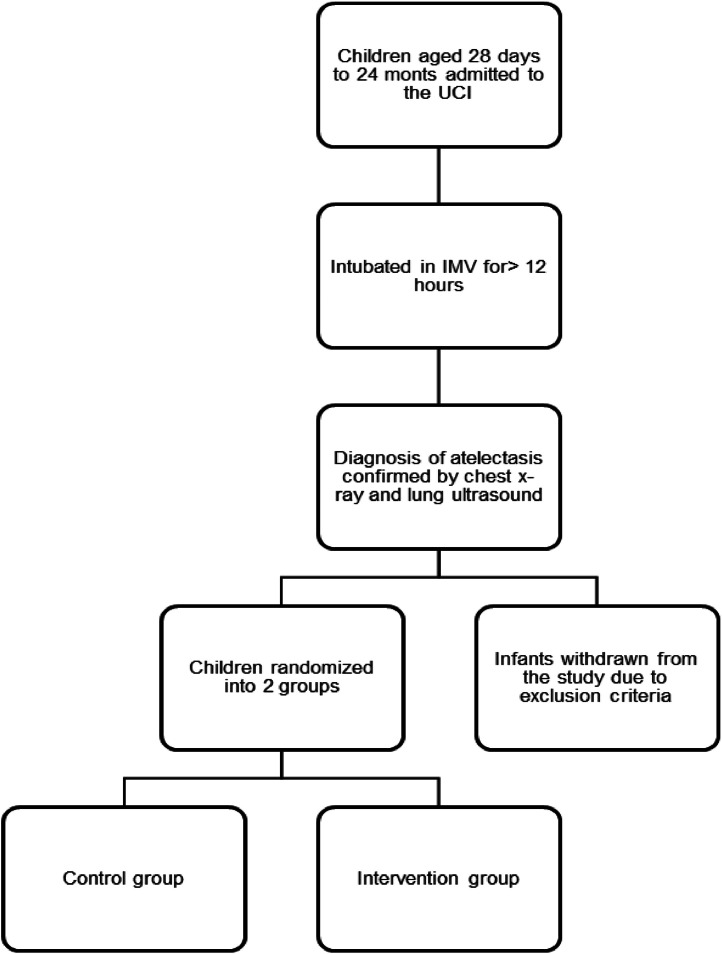
Description of patient selection. Note: ICU, Intensive Care Unit; IMV, Invasive Mechanical Ventilation.

Patients who met the inclusion criteria were electronically randomized (https://www.random.org/lists/) into two groups: Control Group (CG) and Intervention Group (IG) as described in Fig. 1.

The sample size was calculated based on historical audit data from the study site ‒ between August and December 2018, 49 patients underwent tracheal intubation in this PICU, with 13 of these patients developing pulmonary atelectasis. It was therefore estimated that over a period of 12 months, approximately 26‒30 patients would develop pulmonary atelectasis. When entered into a power analysis (using the statistical program openepi.com), with equal numbers in the Intervention (IG) and Control (CG) groups, a sample of 30 patients (15 each in IG and CG) yielded a statistical power of 80%, with 20% error.

In order to characterize the sample, the following data were collected from the patient's medical records: age, gender, clinical diagnosis of hospitalization, weight, height, laboratory tests, and the Pediatric Index of Mortality 2 (PIM 2) outcome prediction score.

Data collection was performed by the physical therapist responsible for the study. Upon detecting the presence of atelectasis on the chest X-Ray, the pediatric intensive care physician responsible for the PICU called the physical therapist via cell phone. After the activation, the researcher checked whether the patient met the inclusion criteria of the study. If so, the authorization of the legal guardian was requested to allow the child's participation in the study through the informed consent form. After signing the informed consent form, the patient was electronically randomized into CG or IG.

### Chest X-ray

The baseline anteroposterior radiological images were considered to be free of atelectasis when there was normal pulmonary transparency with free costophrenic sinuses visualized. Lobar or segmental atelectasis was considered when opacification of the lobe or lung segment was present with any of the following concomitant signs: loss of air/gas volume, the most direct sign being the displacement of the interlobar fissure; the deviation of the heart and mediastinum and the elevation of the diaphragm to the same side as atelectasis in extensive collapse.^18^

The Radiological Atelectasis Scoring System was used to score atelectasis observed on lung X-Rays.^19^ Each radiograph was scored by the physiotherapist for atelectasis, hyperinflation, and mediastinal displacement. The presence or absence of pulmonary hyperinflation was each marked as one (1) point or zero (0) points, respectively. The presence or absence of a mediastinal deviation was scored as one (1) or zero (0). Atelectasis was scored for each lung lobe. Partial atelectasis of one lung lobe was scored as one (1) point, and complete atelectasis of one lobe was scored as two (2) points. The scores were summed for the chest X-Ray of each patient.

### Point of care lung ultrasound (POCLUS)

Lung ultrasound was performed immediately before initiating interventions (baseline) and repeated 30 minutes after intervention (post-intervention measure). The assessment was performed by the main researcher, a physiotherapist trained and certified to perform lung ultrasound. The POCLUS images were recorded and subsequently analyzed by an independent evaluator blinded to study allocation and patient history. The independent evaluator was a pediatric intensive care physician with ultrasonography certification and extensive clinical experience over more than eight years.

The lung was scanned antero-posteriorly, craniocaudally, transversely, and longitudinally to the costal arches. Initially, POCLUS was performed with the patient in the supine position and subsequently turned laterally to evaluate the posterior pulmonary region.

The ultrasound image was considered unaltered when it showed an association between pleural sliding and the presence of horizontal repeats of the pleural line called “A-lines”. A-lines are a type of reflection artifact originating from the pleural line, seen as a series of hyperechoic parallel lines, equidistant from each other, below the pleural line, with spacing equal to the distance between the skin and the pleural line.^12^

The main imaging characteristics of atelectasis visualized by ultrasound are loss of aeration generating a visible, hyperechoic parenchymal area, which may present ill-defined and irregular borders; large lung consolidations with static bronchograms; whilst dynamic air bronchogram can rule out atelectasis.^20^ Other signs of atelectasis on POCLUS include abnormalities in the pleural line and disappearance of the A-line.^21^ The coalescent B-line (or B-pattern) can be interpreted as a higher degree of severity or a state prior to the development of atelectasis, especially if the lesion has a focal location.^22^ The B-lines are hyperechoic vertical artifacts originating from the pleural line that extends to the periphery of the lung field and move with the pulmonary sliding.^23^ At the point of intersection, the B-lines obliterate the A-lines.

The loss of lung aeration identified at POCLUS was graded using the Lung Ultrasound Score (LUS), validated for this purpose.^24^ A score of 0 indicates normal aeration i.e., the presence of lung slippage and horizontal A-lines, or less than three vertical B-lines; a score of 1 indicates moderate loss of aeration indicated by the presence of ≥3 B-lines, regularly or irregularly spaced, originating from the pleural line or small juxta pleural consolidations; a score of 2 indicates severe loss of aeration, i.e.; the presence of coalescing B-lines in several intercostal spaces, occupying the entire intercostal space; and a score of 3 indicates complete loss of pulmonary aeration, characterized by the presence of tissue echogenicity and static or dynamic air bronchograms, as observed in lung consolidation.

### Monitoring

Patients in both groups had vital sign measurements assessed at baseline (immediately before intervention) and at 10 and 30 minutes after intervention: and underwent three evaluations Heart Rate (HR); Respiratory Rate (RR); body temperature; PA; SpO_2_. Clinical severity was classified according to the Wood-Downes Score (WD)^25^ as mild (1 to 3 points), moderate (4 to 7 points) and severe (8 to 14 points) according to the sum of the following items: the presence of wheezing (0 = No; 1 = End of expiration; 2 = Full expiration; 3 = Inhalation and expiration); the presence of chest indrawing (No = 0; Subcostal = 1; Supraclavicular subcostal and nasal flare = 2; supraclavicular, subcostal, intercostal, suprasternal, and nasal flare = 3); total respiratory rate (< 30 breaths per minute = 0; 31‒45 = 1; 46‒60 = 2; > 60 = 3); heart rate (< 120 beats per minute = 0; > 120 = 1); ventilation and breath sound on auscultation (0 = Good and symmetrical breath sounds; 1 = Regular and symmetrical; 2 = Very decreased breath sounds; 3 = Silent chest and cyanosis (0 = No; 1 = Yes).

During monitoring, the number of aspirations, quantity, and quality of aspirated tracheal secretion were recorded, according to the Suzukava Method:^26^ Fluid when the aspiration tube is free of secretions after aspiration, using only vacuum; Moderate, when the aspiration tube presents secretions adhered to the wall of the probe after aspiration but is free after the use of 0.9% saline solution; Thick, when the aspiration probe has secretions adhered to the probe wall even after instillation of 0.9% saline solution.

### Control group

The CG was submitted to routine respiratory physiotherapy care and interventions of the Physical Therapy Service, including manual vibration of the patient's chest wall and Manual Hyperinflation (MH) with a self-inflating bag without control of Peak Inspiratory Pressure (PIP), number of repetitions or established intervals.

The technique of manual vibration in the chest is based on the properties of modifying the consistency of airway mucus. This thixotropic gel, highly viscous under static conditions can become less viscous and is able to flow when shaken.^27^ Thus, when applying vibrations to the chest wall, mechanical energy is transmitted to the airways aiding the ciliary beating, thus reducing the viscosity of bronchial secretions, which can be more easily eliminated by positioning, coughing, or aspiration of the airways.^28^

Manual Hyperinflation (MH) aims to mobilize pulmonary secretions proximally by increasing Peak Expiratory Flow (PEF) and promoting pulmonary re-expansion by increasing pulmonary distension pressure, which favors increased airflow to the poorly ventilated regions through the collateral channels (where present) and by redistributing and renewing surfactant in the alveoli.^29^ The technique is performed by applying a series of deep manual insufflations with brief inspiratory pauses, followed by a rapid release of the bag to increase expiratory flow and stimulate coughing.^28^

### Intervention group

The IG was submitted only to the SRPP developed for this study and applied by the main research physiotherapist. The SRPP intervention included modified postural drainage with mechanical chest wall vibration applied using an electronic massage device (Super da G-Life®); MH using a self-inflating bag; stretching of the respiratory muscles; and functional positioning of the patient in bed.

The patient was first positioned with elevation of the head of the bed by 30° in lateral decubitus, so that the atelectatic pulmonary region was non-dependent, maintaining this position during the application of the other interventions.

Mechanical vibration over the chest wall was performed with the use of a massager positioned over the atelectatic region, in the craniocaudal and lateromedial directions, for ten minutes, with a frequency of 50 Hertz (Hz).^15^ Manual hyperinflation with a self-inflating bag consisted of slow and deep inflation of the bag, followed by an inspiratory pause of two to three seconds and rapid release after this period^29^ with oxygen flow at five liters per minute, with 10 repetitions.^14^ To monitor the PIP provided during MH, a Murenas® analog manometer was used, not exceeding the PIP of 30 cm H_2_O.^29^ No PEEP valve was used.

**Fig. 2:**
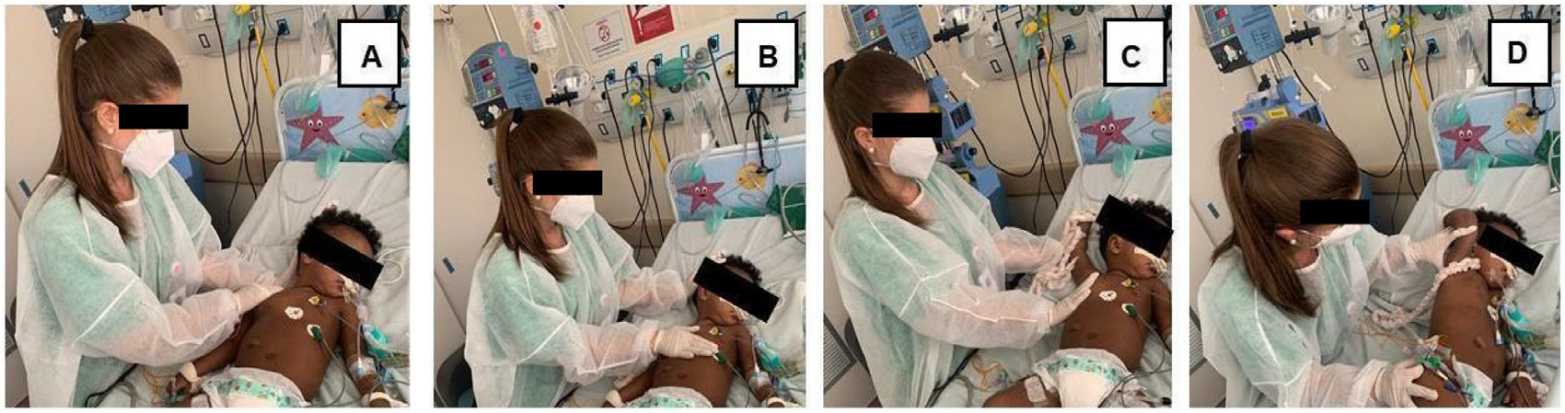
Positioning for stretching the respiratory muscles: (A) Upper trapezius; (B) Sternocleidomastoid; (C) Pectoralis major and (D) Intercostals. Source: The author (2023).

After performing MH, the patients’ accessory respiratory muscles were stretched by the physiotherapist throughout the expiratory phase, bringing the muscle to maximum length, with two sets in 10 consecutive respiratory cycles for each muscle, with a five-second interval between each set.^30^ The stretches were performed bilaterally as follows: upper trapezius: with the patient positioned in the dorsal decubitus position, the physiotherapist rested one hand on the occipital region, side flexing the head to the opposite side whilst, with the other hand, moving the ipsilateral shoulder in the craniocaudal direction; sternocleidomastoid: with the patient positioned in the dorsal decubitus position, the physiotherapist passively flexed (away from the muscle to be stretched) and laterally rotated towards the target muscle, by placing one hand in the occipital region and the other on the upper thorax region, displacing in the craniocaudal direction; pectoralis major: with the patient positioned in dorsal decubitus, with the arm to be stretched abducted and externally rotated at the shoulder, with elbow flexion, the physiotherapist applied a passive stretch by applying pressure using one hand on the upper third of the arm and the other on the lateral region of the upper thorax, following the orientation of the muscle fibers; intercostal muscles: with the patient in lateral decubitus with the forearm flexed and the hand resting on the occiput, the physiotherapist supported the patient's arm with one hand while the other was positioned on the lower rib cage during inspiration; the physiotherapist facilitated expansion of the rib cage by moving the patient's arm in the craniocaudal direction and following the expiratory movement without applying pressure (Fig. 2).^30^

In both groups, the orotracheal tube was aspirated, when necessary, after the interventions and during the procedures. The quantity and quality of aspirated tracheal secretion were classified using the Suzukava method.^26^

Finally, functional positioning was performed in bed, aiming to favor respiratory mechanics, optimize pulmonary function, and stimulate the child's sensorineural and psychomotor development.

### Interrupt criteria

The criteria for interruption of the protocol were: Heart Rate (HR) greater than 200 beats per minute (bpm); Respiratory Rate (RR) greater than 45 breaths per minute (bpm); Blood Pressure (BP) values above 120/80 millimeters of mercury (mmHg) or less than 80/40 mmHg; Pulse Oxygen Saturation (SpO_2_) less than 88% with the need for increased FiO_2_ during the application of the protocol. The presence or absence of signs of respiratory distress (accessory respiratory muscle use, pallor, sweating, and psychomotor agitation) was also considered according to the Wood-Downes Score (WD) used in this study.^26^ In the presence of any changes mentioned above, the protocol was interrupted, and the child was placed in bed and kept under monitoring. The patient who needed to interrupt the procedures could be included again in the study six hours after interrupting the first attempt. Daily attempts could be made, within 48 hours of the first attempt, after this period the patient was considered as not benefiting from the study protocol.

### Statistical analysis

Descriptive statistics included measures of central tendency by means ± Standard Deviations (SD), medians and interquartile ranges (IQR 25%‒75%), and absolute and relative frequencies.

The Kolmogorov-Smirnov test was used to evaluate the normality of the distribution, and considering that most variables were not normally distributed, between-group analyses were conducted using the non-parametric Wilcoxon/Mann-Whitney tests. Fisher's exact tests were used for comparisons between frequencies. To compare repeated vital sign measurements (before physical therapy, after 10 and 30 minutes), the non-parametric Friedman test was used, with post-hoc analysis using the paired Wilcoxon test (signed rank). Bonferroni correction was used as appropriate.

### Evaluation of the magnitude of the effect (effect size)

The intervention of the study was not compared to a placebo, but to a control group, in which routine physical therapy of the Hospital was performed. Therefore, statistical differences were not expected in the two groups when comparing parameters before and after respiratory therapy, and the only way to evaluate the outcome of the study intervention is through the magnitude of the effect (effect size). The standardized effect magnitude allows researchers to communicate the practical significance of the results, rather than just reporting statistical significance.^31^ Cohen's “*d*” test (Cohens' d) is used to describe the standardized mean difference of an effect. A correction of Cohens' d is Hedges' “*g*” (Hedges' g), which is unbiased and corrected for small samples (n < 20), and it was this test that the authors used to measure the effect of the intervention on the LUS and Wood Downes scores (paired measures before and after respiratory physiotherapy). The way to interpret the Hedges' g is as suggested by Cohen: small (0.2 < Hedges' g < 0.5), moderate (0.5 < Hedges' g < 0.8), and large (Hedges' g ≥ 0.8 effects).^31^ These values assume negative values when the effect is reduced, e.g., a reduction in a score; but they can be informed by their absolute values. If the absolute value is greater than one (1), it means that the difference between the means is greater than one SD.

The analyses were performed using R: A language and environment for statistical computing. R Foundation for Statistical Computing, Vienna, Austria. URL https://www.R-project.org/.

## Results

**Fig. 3:**
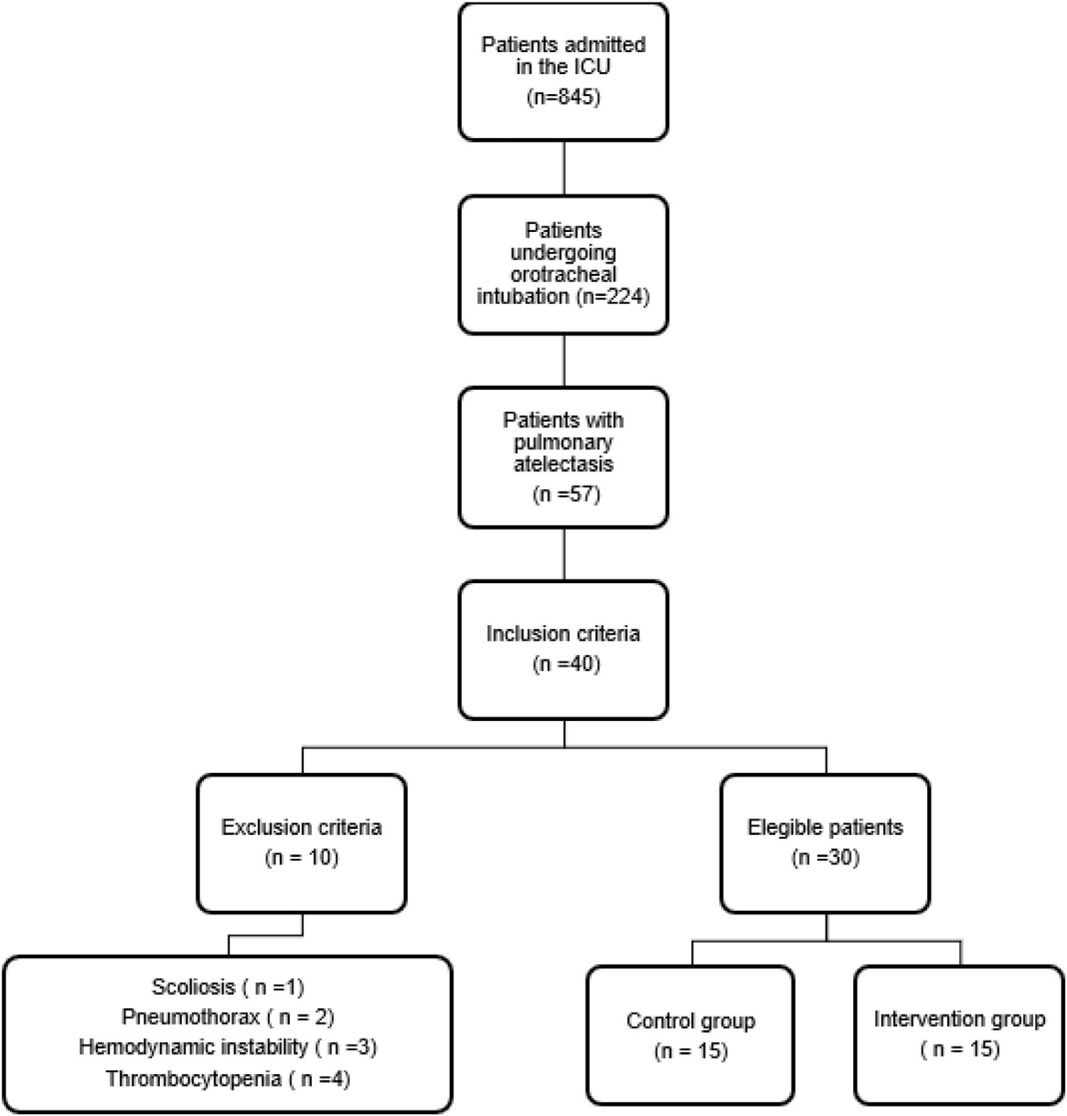
Sample selection flowchart.

During the study period, 845 children were admitted to the PICU of the HMIMJ, of which 26% underwent orotracheal intubation and 7% developed pulmonary atelectasis. Of these children, 40 met the inclusion criteria due to atelectasis, 10 patients were excluded (one with scoliosis, two with orotracheal tube displacement, tissue trauma, air leak syndrome (pneumothorax or pneumomediastinum) (Fig. 3).

The sample of 30 children was randomized into two groups (n = 15 in the CG; n = 15 in the IG). The median (IQR 25%‒75%) age of the patients was 7 (2‒17) months in the CG and 4 (2‒13.5) months in the IG. The groups presented similar characteristics (p > 0.05) in terms of epidemiology and clinical variables (Table 1).

**Table 1:** Epidemiological characteristics, clinical variables and laboratory tests of the sample according to groups and results of statistical tests.

Variables	Group Control		Group Intervention		p*
N	15		15		‒
Female gender, n (%)	6	40	4	26.7	0.6
Age (months)^a^	7	2.0‒17.0	4	2.0‒13.5	0.7
Diagnostics					
Viral bronchiolitis	11	73.3	11	73.3	^c^
Pneumonia	‒	‒	1	6.7	^c^
Nephrotic syndrome	‒	‒	1	6.7	^c^
Septic shock	3	20	2	13.3	^c^
Wheezing crisis	1	6.7	‒	‒	^c^
Weight (Kg)^b^	7.7	3.2	7.4	2.9	0.4
Height (cm)^b^	68.3	12	67	12.6	0.7
BMI (Kg/cm^2^)^b^	15.5	1.7	16.2	1.6	0.4
PIM2^b^	5	7.5	2.1	1.3	0.6
Duration of IMV (days)^a^	7	5.5‒8.5	6	4.5‒8.5	0.5
ICU length of stay (days)^a^	15	11.0‒16.0	12	9.5‒18.5	0.4
Hospital length of stay (days)^a^	19	17.0‒24.0	21	12.5‒24.5	0.6
pH^a^	7.4	0.1	7.4	0.1	0.3
PaO_2_^a^	91.5	40.3	108.7	37.8	0.3
PaCO_2_^a^	46	10.7	46.3	14.1	0.8
HCO_3_^a^	29	7	26.7	5.2	0.2
BE^a^	3.8	7.3	1.1	4.9	0.1
SaO_2_^a^	93.1	8.3	96.3	4.5	0.5
PaO_2_/FiO_2_^a^	256.9	171.4	299.7	106.6	0.3
Hb^a^	10	1.4	9.7	1.5	0.3
Ht^a^	30.2	4.2	27.6	4.5	0.07
Platelets^a^	326533.3	143116.8	316000	134982	0.6
Lactate^a^	1.8	1.6	1.6	1	0.9
CRP^a^	3.5	2.9	5.7	5.5	0.4
Viral panel					
RSV	9	81.8	8	72.7	^c^
Bocavirus	2	18.2	‒	‒	^c^
Parainfluenza I e II	‒	‒	1	9.1	^c^
Metapnemovirus	‒	‒	1	9.1	^c^
Seasonal coronavirus	‒	‒	1	9.1	^c^

NOTE: ^a^ Median (IIQ 25%‒75%); ^b^ Mean ± Standard Deviation; ^c^ Variables with multiple subvariables did not allow generating statistical significance due to the number of cases.

p*, p-values by the Wilcoxon/Mann-Whitney test or Fisher's exact test, when applicable; N, Absolute number; Kg, Kilogram; cm, Centimeters; BMI, Body Mass Index; PIM2, Pediatric Index of Mortality 2; IMV, Invasive Mechanical Ventilation; ICU, Intensive Care Unit, pH, Hydrogen Potential; PaO_2_, Partial Pressure of Oxygen; PaCO_2_, Partial Pressure of Carbon Dioxide; BE, Base Excess; SaO_2_, Arterial Oxygen Saturation; PaO_2_/FiO_2_, Ratio of Partial Pressure of Oxygen to the fraction of inspired oxygen; Hb, Hemoglobin; Ht, Hematocrit; CRP, C-Reactive Protein; RSV, Respiratory Syncytial Virus.

Laboratory parameters prior to respiratory therapy interventions were statistically similar (p > 0.05), see Table 1. Ventilatory parameters and Radiological Atelectasis Scoring System scores of both groups were similar (p > 0.05), see Table 2. Regarding the variables diagnostics, viral panel, IMV mode, VAP, location of atelectasis, quality and color of secretion, it was not possible to add statistical significance due to the small sample size in the subcategories of each of them.

**Table 2:** Description of modes, ventilation parameters, incidence of Ventilator-Associated Pneumonia (VAP), Radiological Atelectasis Scoring System score, location of atelectasis and characteristics of tracheal secretion according to groups and results of statistical tests.

Variables	Control group		Intervention group		p*
IMV mode, n (%)					
PCV (cm H_2_O)	11.0	73.3	10.0	66.6	^b^
PSV (cm H_2_O)	1.0	6.7	1.0	6.7	^b^
PRVC (cm H_2_O)	‒	‒	1.0	6.7	^b^
SIMV (cm H_2_O)	3.0	20.0	3.0	20.0	^b^
Ventilatory parameters^a^					
PIP (cm H_2_O)	21.7	3.0	22.1	3.6	0.8
PEEP (cm H_2_O)	7.0	1.0	6.8	0.6	0.7
RR (ipm)	27.3	5.3	25.8	3.8	0.7
IT (s)	0.6	0.1	0.6	0.1	0.7
FiO2 (%)	44.0	18.4	37.8	12.2	0.6
MAP (cm H_2_O)	11.5	2.0	13.0	1.9	0.1
TV (mL)	65.8	36.8	61.3	38.9	0.3
VT (mL/Kg)	6.0	3.0	8.0	2.5	0.9
Intratracheal cuff, n (%)	6.0	40.0	6.0	40.0	^b^
VAP, n (%)	2.0	13.3	1.0	6.7	^b^
Radiological score^a^	2.7	0.9	3.1	0.9	0.3
Location of atelectasis^a^					
RUL	9.0	60.0	11.0	73.3	^b^
RML	4.0	26.7	4.0	26.7	^b^
LLL	2.0	13.3	‒	‒	^b^
Aspirations^a^	1.8	0.8	2.1	0.8	0.4
Quality, n (%)					
Moderate	14.0	93.3	15.0	100.0	^b^
Thick	1.0	6.7	‒	‒	^b^
Colour, n (%)					
Clear	10.0	66.7	10.0	66.7	^b^
Yellowish	5.0	33.3	5.0	33.3	^b^

Note: ^a^ Mean and standard deviation; ^b^ Variables with multiple subvariables did not allow generating statistical significance due to the number of cases.

p*, p-values by the Wilcoxon/Mann-Whitney test.

N, Absolute Number; IMV, Invasive Mechanical Ventilation; PCV, Pressure Controlled Ventilation; PSV, Pressure Support Ventilation; PRVC, Pressure-Regulated and Volume-Controlled Ventilation; SIMV, Synchronized Intermittent Mandatory Ventilation; cm H_2_O, Centimeters of Water; PIP, Peak Inspiratory Pressure; PEEP, Positive End-Expiratory Pressure; RR, Respiratory Rate; ipm, Incursions per minute; IT, Inspiratory Time; s, seconds; FiO_2_, Fraction of Inspired Oxygen; MAP, Mean Airway Pressure; TV, Total Volume; mL, Milliliter; VT, Tidal Volume; mL/Kg, Milliliter per kilo; VAP, Ventilator-Associated Pneumonia; Radiological score, Radiological Atelectasis Scoring System; RUL, Right Upper Lobe; RML, Right Middle Lobe; LLL, Left Lower Lobe.

### Evaluation of lung ultrasound score (LUS) and Wood-Downes (WD) scores

There was no significant difference in the baseline or post-intervention median (IQ 25%‒75%) LUS scores between the control and intervention groups: 2 (1‒3) vs. 3 (2‒3) (p = 0.21) and 1 (1‒2.5) vs. 1 (0.5‒2) (p = 0.5) respectively. Similarly, there were no significant between-group differences in the median (IQ 25%‒75%) baseline or post-intervention WD scores between CG and IG: 4 (3‒5) vs. 4 (3‒5) (p = 0.9) and 3 (2‒3) vs. 2 (1–2.5) (p = 0.18), respectively.

Significant within-group differences were observed in both CG and IG comparing baseline to post-intervention measures (Table 3). Median (IQR 25%‒75%) LUS in the CG and IG changed from 2(1–3) and 3 (2–3) before respiratory physiotherapy to 1 (1–2.5; p = 0.01) and 1 (0.5–2; p < 0.001) after intervention respectively. Median (IQ 25%–75% WD score changed from 4 (3.5‒5) and 4 (3–5) to 3(2–3; p < 0.001) and 2 (1–2.5; p < 0.001) postintervention in the control and intervention groups respectively.

**Table 3:** Description of LUS and Wood-Downes Score comparing groups, moments and results of statistical tests.

Score	Median	P 25	P 75	p*
LUS CG before	2.0	1.0	3.0	0.01
LUS CG after	1.0	1.0	2.5	

LUS IG before	3.0	2.0	3.0	<0.001
LUS IG after	1.0	0.5	2.0	

WD CG before	4.0	3.5	5.0	<0.001
WD CG after	3.0	2.0	3.0	

WD IG before	4.0	3.0	5.0	<0.001
WD IG after	2.0	1.0	2.5	

Note: P25, 25^th^ percentile; P75, 75^th^ percentile.

p*, p-values by the Wilcoxon/Mann-Whitney test.

LUS, Lung Ultrasound Score; WD, Wood-Downes; CG, Control Group; IG, Intervention Group; Before, Before respiratory physiotherapy; After, After respiratory physiotherapy.

### Assessment of the magnitude of the effect

**Fig. 4:**
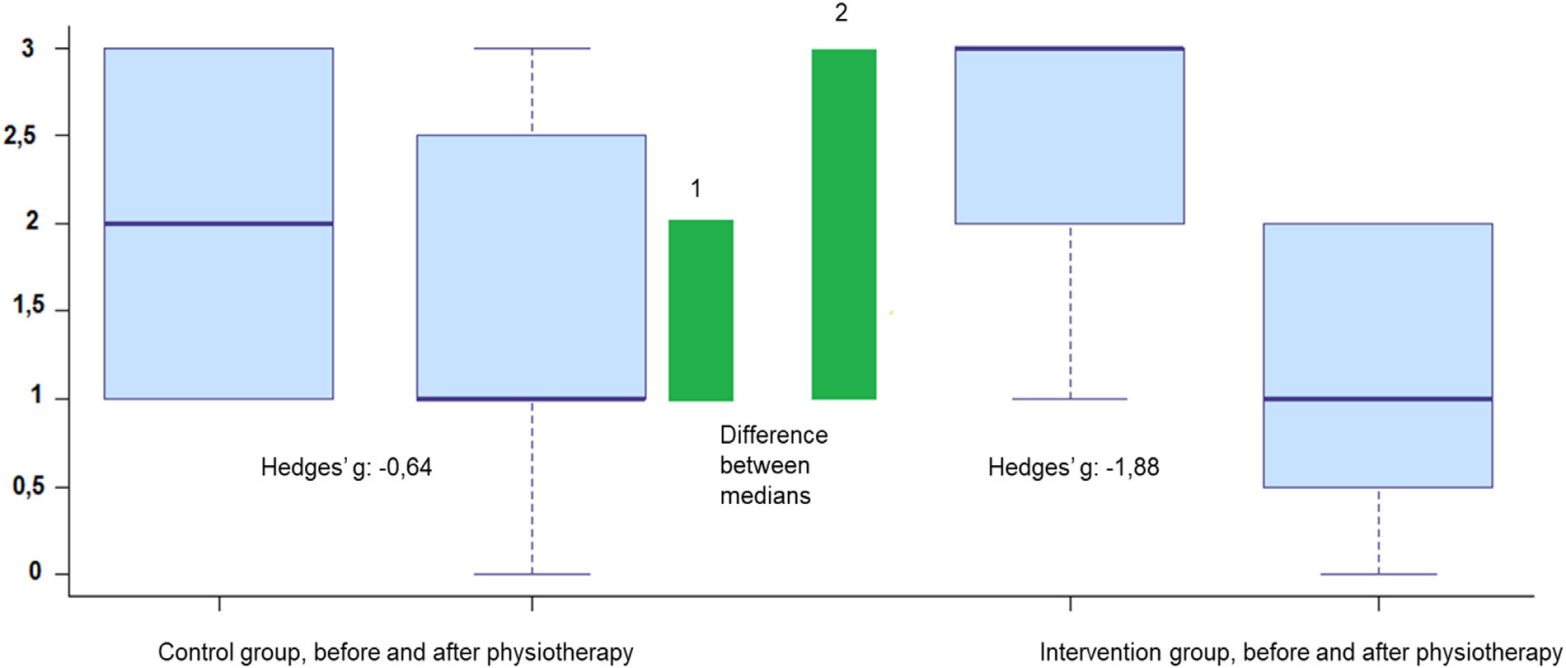
Magnitude of the effect (effect size) of the intervention proposed in the study (GI) on LUS (y axis), compared to the effect of routine physiotherapy (CG), shown by the differences between the medians (green bars) and the values “g” for Hedges.

There was a moderate effect on the reduction of the LUS score in the CG after respiratory physiotherapy (Hedges' g = -0.64, 95% CI: -1.35 to 0.08), and a 2.9-fold greater effect on the IG (Hedges' g = -1.88, 95% CI: -1.01 to -2.73), characterizing a large effect on the reduction of this score (Fig. 4).

**Fig. 5:**
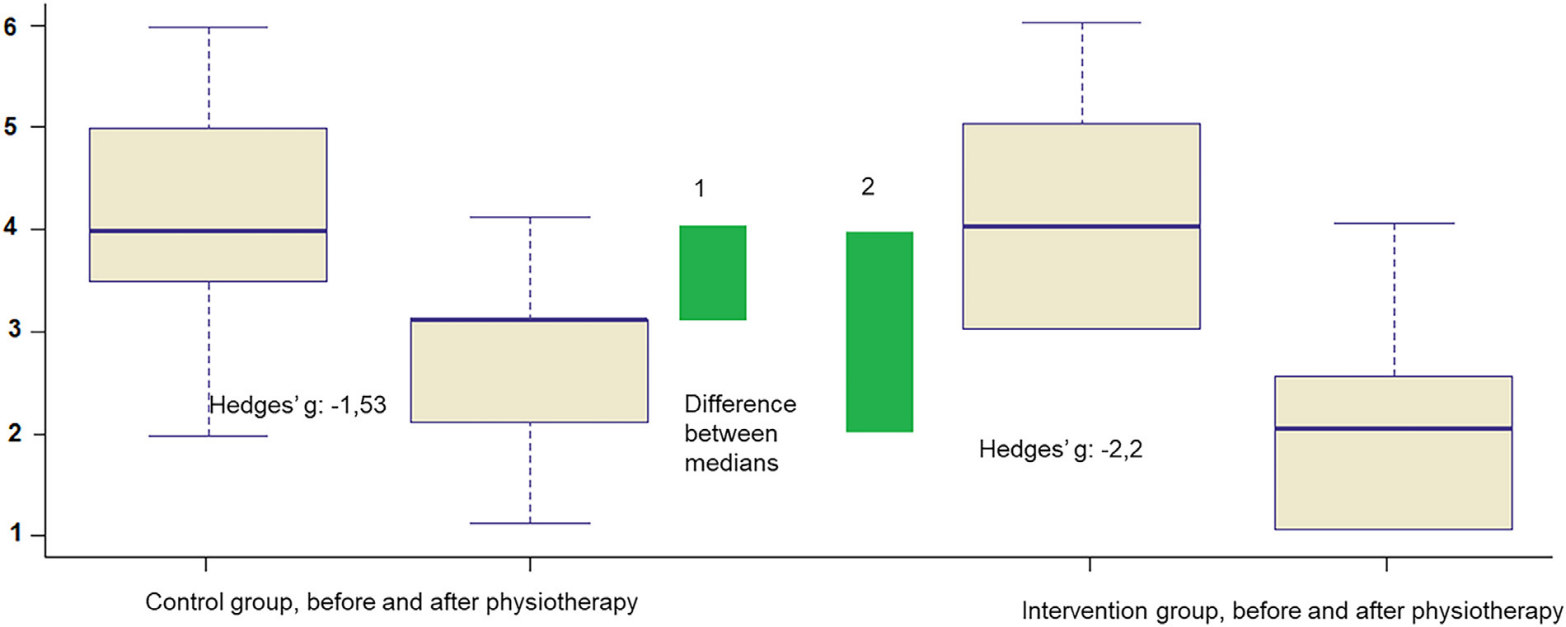
Magnitude of the effect (effect size) of the intervention proposed in the study (GI) on the Wood-Downes score (y-axis), compared to the effect of routine physiotherapy (CG), shown by the differences between the medians (green bars) and by Hedges’ “g” values.

There was a large effect on the reduction of the WD score in the CG after physical therapy (Hedges' g = -1.53, 95% CI -3.1 to -1.29), and a 1.4-fold greater effect in the IG (Hedges' g = -2.2, 95% CI: -2.32 to -0.71) (Fig. 5).

### Evaluation of effects on physiological parameters

The parameters of heart rate, respiratory rate, temperature, SpO_2_, systolic, mean, and diastolic blood pressure at all measurement points were statistically similar between CG and IG (Table 4).

**Table 4:** Description of vital signs according to groups and moments and results of statistical tests.

Variables	Group control		Group intervention		p*
HR before (bpm)	139.9	26.7	142.7	23.3	0.9
HR 10 min (bpm)	140.9	22	140.5	20.3	0.8
HR 30 min (bpm)	137.1	23.9	135.9	19.7	0.8
RR before (ipm)	38.3	11.1	37.4	10.2	0.8
RR 10 min (ipm)	36.8	8.5	34.7	7.6	0.7
RR 30 min (ipm)	32.5	7.1	32.3	5.4	0.9
Temp. before (°C)	36.7	0.5	36.6	0.5	0.7
Temp. 10 min (°C)	36.6	0.4	36.6	0.5	0.7
Temp. 30 min (°C)	36.7	0.4	36.6	0.5	0.6
SPO_2_ before (%)	95.5	3.4	96.5	4.1	0.4
SPO_2_ 10 min (%)	98.2	2.5	98.2	2	0.8
SPO_2_ 30 min (%)	97.9	2.4	98.5	2.2	0.5
SBP before (mmHg)	98.1	17.2	104.3	13.1	0.3
SBP 10 min (mmHg)	104.5	17.3	104.1	15.4	0.8
SBP 30 min (mmHg)	99.4	20.5	98.5	16.6	0.6
DBP before (mmHg)	57.1	14.3	59.5	9.6	0.4
DBP 10 min (mmHg)	57.6	12.5	53.7	10.2	0.7
DBP 30 min (mmHg)	55.4	13.5	52.9	11.5	0.7
ABP before (mmHg)	71.5	14.3	75.5	10.1	0.3
ABP 10 min (mmHg)	74.4	13	71.6	12.6	0.6
ABP 30 min (mmHg)	71.2	13.9	68	12.2	0.3

Note: Data are in means and standard deviations.

p*, p-values according to the non-parametric Friedman test, with post-hoc analysis using the paired Wilcoxon test (signed rank), with Bonferroni correction.

Before, Before respiratory physiotherapy; HR, Heart Rate; min, Minutes; RR, Respiratory Rate; Temp, Body Temperature in degrees Celsius; degrees Celsius; SPO_2_, Peripheral Oxygen Saturation; SBP, Systolic Blood Pressure; DBP, Diastolic Blood Pressure; ABP, Average Arterial Pressure.

## Discussion

The loss of lung volume caused by atelectasis can modify respiratory mechanics and impair gas exchange, which can lead to an increase in IMV time, tracheal extubation failure, increased length of hospital stays, and morbidity and mortality.^32^ This study, including children undergoing IMV, evaluated the effectiveness of a Structured Respiratory Physical Therapy Protocol (SRPP) by means of immediate evaluation by pulmonary ultrasonography compared to a control group aimed at airway clearance and pulmonary re-expansion of atelectasis areas.

This is not the first study to report the use of physiotherapist-applied non-invasive interventions to treat pulmonary atelectasis in children on IMV. However, this is the first study to develop and evaluate an SRPP for the resolution of unilateral pulmonary atelectasis in children.

In both intervention and control groups, the most frequent location of pulmonary atelectasis was the right upper lobe, which has been described previously.^15^, ^16^ This finding can be explained anatomically by the fact that the right upper lobe bronchus is at a 90-degree angle from the right main bronchus, limiting secretion drainage.^33^

As described in the study by Galvis et al.,^14^ the factors that may have contributed to the development of atelectasis in these patients in addition to the anatomical and physiological features typical of the pediatric age group include factors related to the health status and critical care modalities. These may include alteration of mucociliary transport, resulting from the artificial airway, mucosal edema and/or excessive mucus production due to trauma associated with repeated suctioning; thickening of mucus caused by the disease process, fluid restriction and diuretic use; accumulation of secretions resulting from inadequate bronchial drainage, particularly in children receiving excessive sedation and neuromuscular blockade; inadequate humidification of inspired gas and incomplete removal of tracheal secretions during tracheal aspiration.^34^

Previous studies^14^, ^15^, ^16^, ^17^ on this topic have used chest X-Rays as the primary tool to evaluate the resolution of pulmonary atelectasis and the efficacy of treatments. By offering diagnostic accuracy similar to chest radiography, without exposure to ionizing radiation, POCLUS is a dynamic and agile tool to perform and interpret lung changes, quickly integrating the information into the patient's clinical context.^35^ Ultrasound was successfully used in this study to evaluate the outcome of respiratory physiotherapy interventions performed in children on IMV with pulmonary atelectasis, which was detected in the first evaluation by means of radiological imaging, evaluated by the intensive care physician.

Bedside lung ultrasound is a diagnostic tool that has been increasingly used in Intensive Care Units (ICU), because it is a safe test for both the patient and the team, and can be performed frequently at the bedside by adequately trained PICU professionals, including physiotherapists, allowing non-invasive monitoring of progression and response to therapeutic interventions accurately, quickly, safely and dynamically.^35^ Although the authors cannot comment on the sensitivity, specificity or reliability of POCLUS in this context, as post-intervention images were not compared with chest X-Ray findings, owing to ethical and resource limitations, and between-rater comparison was not made, previous studies have reported that lung ultrasound is highly reliable both sensitive and specific in identifying and quantifying pathological changes, including atelectasis.^12^, ^21^ Although further studies are needed, this study supports the feasible and potential utility of using POCLUS to determine short-term responses to chest physiotherapy interventions.

In addition to the evaluation, the LUS^24^ was used to quantitatively classify the POCLUS image before and after respiratory physiotherapy interventions in both groups. Adult and neonatal clinical practice experts have suggested that clinical evaluation plus semi-quantification of ultrasound scores can be used as a tool to quantify peripheral lung aeration and clinical severity of the patient.^36^ This approach is based on the hypothesis that the lower the peripheral lung expansibility (areas visualized by the POCLUS), the fewer lung areas will be available for gas exchange.^37^ Therefore, it is expected that the clinical manifestations of these pulmonary alterations will be more severe.

Faced with this hypothesis, a retrospective study,^37^ including 74 children from zero to 12 months of age, diagnosed with bronchiolitis, and admitted to PICUs, developed a simple and rapid score that combines clinical data (presence of wheezing and reduced oral intake) and ultrasound data (involvement of the right posterior upper pulmonary zone) to predict, during the first evaluation, the need for hospitalization in the PICU, as well as the indication of ventilatory support with CPAP (continuous airway pressure).

However, one of the limitations of the study was the absence of patients on IMV.

In the present study, although there were large within-group effect sizes, no significant between-group differences were observed in the clinical (WD)^25^ or Ultrasonographic (LUS)^24^ scores before versus after respiratory physiotherapy intervention. This suggests that both IG and CG interventions were associated with an improvement in pulmonary imaging (partial or total resolution of pulmonary atelectasis) and in the children's breathing patterns. The magnitude of the treatment effect was, however, greater in the intervention group, suggesting that the protocolized intervention may be more effective at resolving atelectasis in mechanically ventilated children. This requires confirmation in a larger sample study.

The application of mechanical thoracic vibration in the IG may have been one of the factors related to the better findings in the reduction of WD and LUS scores in this group. The natural frequency of ciliary beats in mammals is 13 Hz, an increase in bronchial clearance is observed when vibrations reach frequencies between 11 and 15 Hz.^38^ In the range between 20 and 45 Hz, vibration produces relaxation of the respiratory muscles, verified by a decrease in respiratory rate and an increase in tidal volume.^38^ The mechanical vibration apparatus used in the study provides a continuous frequency of 50 Hz and, therefore, may have been one of the factors related to the better findings in the intervention group. Bilan et al.^15^ used mechanical vibration in some patients in their study for 10 to 20 minutes, and there was no comparison between the interventions or the description of the frequency in Hz used.

Another intervention included in the SRPP, which may have influenced the resolution of pulmonary atelectasis, is the stretching of the accessory respiratory muscles. The stretching of a muscle fiber promotes an increase in the number of sarcomeres in series. The addition of muscle strength due to stretching is possibly due to the better interaction between actin and myosin filaments.^39^ Thus, the stretching of the respiratory muscles may have helped in the better performance of the inspiratory and expiratory muscles and increased thoracic expansion and may have contributed to the improvement of respiratory mechanics and to an increase in lung volumes.

Another intervention included in the SRPP, which may have contributed to better results in the intervention group, is manual hyperinflation with a self-inflating bag performed in a standardized manner. Regarding the form of MH application, only one study^17^ explained how the intervention was applied (first, slow insufflation, followed by an inspiratory pause of two to three seconds, followed by rapid pressure release), and was performed in the same way in the present study, with the objective of promoting an increase in Peak Expiratory Flow (PEF), displacing secretion to the central airways and simulating the effect of coughing.^29^^,^^40^ As a safety measure to avoid baro and volutrauma, both the present study and the studies by Galvis et al.^14^ and Herrada et al.^17^ used a manometer during MH to limit Peak Inspiratory Pressure (PIP) between 30‒35 cm H_2_O.

Regarding the safety of the interventions proposed by the SRPP, vital signs are important, as they allow the rapid identification of clinical deterioration of patients before, during, or after physiotherapy interventions.^29^ In the present study, vital signs in both groups were statistically similar before and after respiratory physiotherapy. It was not necessary to interrupt the interventions in any of the groups, demonstrating that they do not cause risks of clinical deterioration (bradycardia or tachycardia, tachypnea, drop in SpO_2_ or changes in blood pressure) to the patients included in the study, being safe in this context. The retrospective study by Herrada et al.^17^ also reported that the respiratory physiotherapy interventions were well tolerated and that although all patients presented with tachycardia after the interventions, none of them presented with significant hemodynamic consequences that required medical intervention.

Among the limitations of this study are: a) The SRPP was performed only once, and the patient was not followed up after 30 minutes. Therefore, it was not possible to evaluate maintenance of any clinical or ultrasound improvements, nor the impact on meaningful clinical outcomes such as duration of IMV or PICU length of stay; b) The US findings were not compared to chest X-Ray, so you cannot comment on sensitivity or specify or the tool in this context; c) Variables with several sub-variables did not allow comparison due to the number of cases; d) Due to COVID-19, one of the PICUs in the institution where the study took place was closed, due to the low demand of pediatric patients during this period, making it possible to reach the planned sample size of the study, but over a longer period of data collection.

There are still challenges to be faced for the implementation of POCLUS in the routine of physical therapists, such as the provision of skills training, mentoring, and support from experienced mentors. It is a tool that can optimize the functional diagnosis made by the physiotherapist, as well as guide the interventions that may be proposed. Further research is needed to identify the impact of the inclusion of ultrasound in the clinical decision-making of physiotherapists.

It is suggested that SRPP be applied to larger samples and with longitudinal follow-up to confirm its benefits in the medium and long term, as well as to compare component interventions to identify which one has the greatest impact on the resolution of pulmonary atelectasis in children on IMV.

## Conclusion

This study has shown that the Structured Respiratory Physical Therapy Protocol appears to be safe and may be effective in improving airway clearance and lung re-expansion in children on IMV with unilateral pulmonary atelectasis.

## Conflicts of interest

The authors declare no conflicts of interest.
